# Drivers of diversification in fungal pathogen populations

**DOI:** 10.1371/journal.ppat.1012430

**Published:** 2024-09-12

**Authors:** Daniel Murante, Deborah Ann Hogan

**Affiliations:** Department of Microbiology and Immunology, Geisel School of Medicine at Dartmouth, Hanover, New Hampshire, United States of America; University of Georgia, UNITED STATES OF AMERICA

## Abstract

To manage and treat chronic fungal diseases effectively, we require an improved understanding of their complexity. There is an increasing appreciation that chronic infection populations are often heterogeneous due to diversification and drift, even within a single microbial species. Genetically diverse populations can contribute to persistence and resistance to treatment by maintaining cells with different phenotypes capable of thriving in these dynamic environments. In chronic infections, fungal pathogens undergo prolonged challenges that can drive trait selection to convergent adapted states through restricted access to critical nutrients, assault by immune effectors, competition with other species, and antifungal drugs. This review first highlights the various genetic and epigenetic mechanisms that promote diversity in pathogenic fungal populations and provide an additional barrier to assessing the actual heterogeneity of fungal infections. We then review existing studies of evolution and genetic heterogeneity in fungal populations from lung infections associated with the genetic disease cystic fibrosis. We conclude with a discussion of open research questions that, once answered, may aid in diagnosing and treating chronic fungal infections.

## Introduction

Chronic or long-term fungal infections are either not readily cleared by the host or never eliminated, often despite antifungal therapy. Most fungal infections are caused by opportunistic pathogens that establish infections in individuals with reduced host defenses, including primary or secondary immunodeficiencies due to AIDS, diabetes mellitus, Coronavirus Disease 2019 (COVID-19), chronic granulomatous disease, immunosuppressive therapies or prolonged antibiotic treatment or defects in microbial clearance such as that associated with the genetic disease cystic fibrosis (CF) [1–9]. *Candida* spp., *Cryptococcus neoformans*, and *Aspergillus fumigatus* are the most common agents of fungal infections, though the specific incidence of chronic infections caused by these pathogens is less clear. Chronic fungal infections associated with endemic mycoses [10–13] include those caused by *Histoplasma capsulatum*, *Blastomyces dermatitidis*, and *Coccidioides immitis*, which can endure for long periods as asymptomatic or mild presentations before advancing to more damaging disease states.

Improvements in microbial profiling technologies have led to the recognition that microbial diversity exists over space and time and at multiple scales, from polymicrobial communities to within a population and even between genetically identical cells. This review will first focus on mechanisms by which genetic diversity increases within fungal populations [14] and examples of how each mechanism has been shown to contribute to diverse fungal infections. Ecological theory has long recognized the intrinsic value of biodiversity on the function and stability of an ecosystem, referred to as “biological insurance theory” [15]. Therefore, the stability of a diverse microbial ecosystem presents a dilemma when treating bacterial or fungal infections comprised of heterogeneous subpopulations. An inability to evaluate diversity within an infection may lead to the use of therapeutic strategies that allow for the survival of treatment-resistant lineages that can reestablish the infection [16]. Thus, there is a growing need to apply ecological theory to population-level genomic data to predict cases in which problematic subpopulations may be present.

An additional limiting factor in the development of this field of research is that genetic heterogeneity in the context of chronic fungal infection is generally not currently evaluated diagnostically. With the increased capacity for next-generation sequencing of multiple isolates or populations from clinical samples, we now have the potential to catalog population heterogeneity and microbial succession comprehensively. Analyzing population-level heterogeneity in chronic infections of various fungi and the common themes apparent in infections in multiple individuals can provide insight into selective pressures that drive evolution in vivo and may indicate pathways to target for therapy development. The second half of the review will give special attention to fungal diversification and evolution studies in the context of chronic CF infections. We will discuss how the analysis of these populations has informed us about factors within the host environment that drive selection, such as drug treatment, host immune factors, nutrient restriction, and reactive compounds. We will conclude with a discussion of open questions regarding the causes and consequences of heterogeneity in chronic infections. In the future, our understanding of the fungal subpopulations present in chronic infections may allow for a more precise approach to their treatment [17].

### Mechanisms that contribute to the generation of biologically relevant heterogeneity in fungal populations

**Fig 1:**
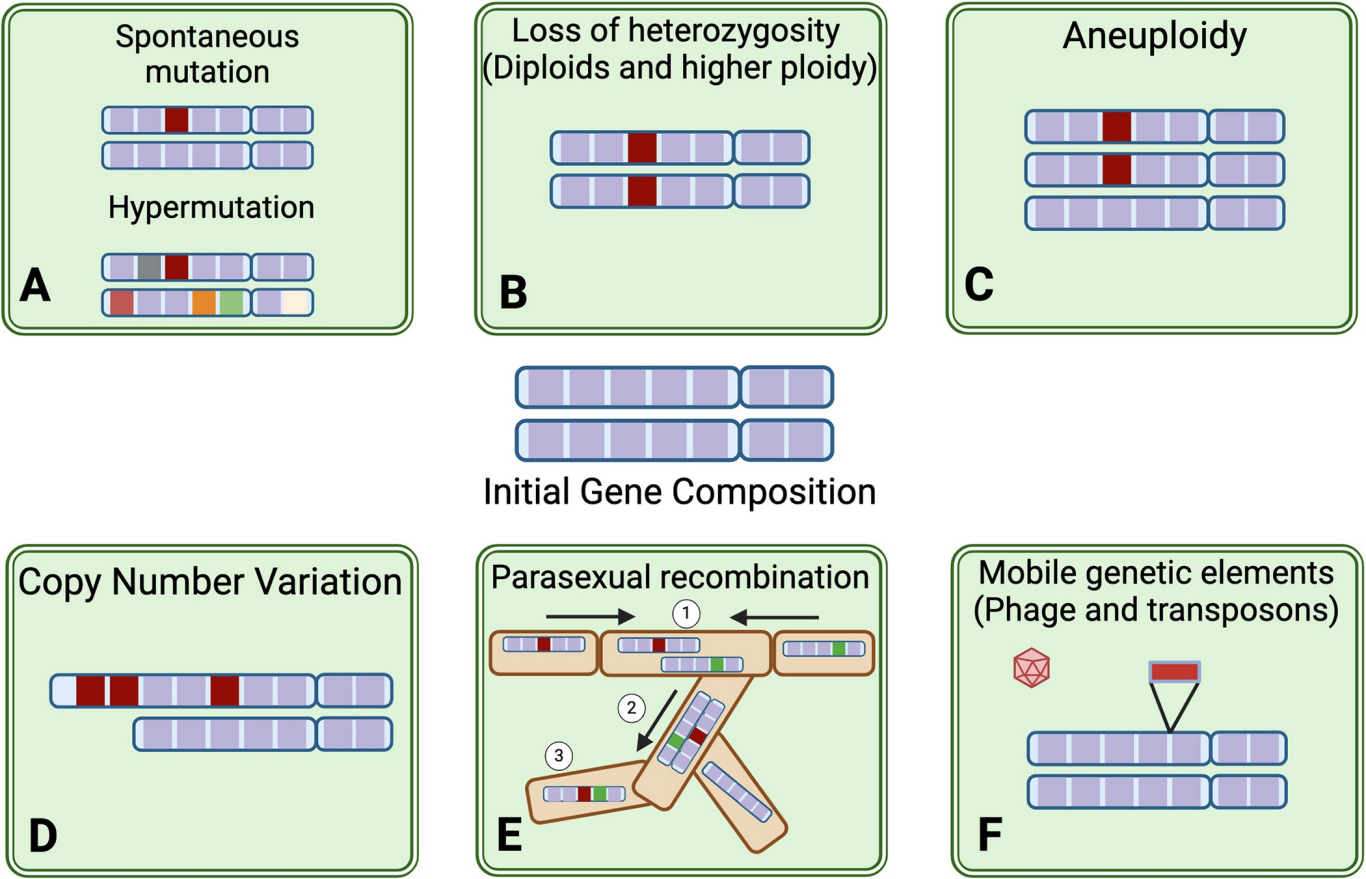
Genetic mechanisms leading to phenotype diversification in fungal pathogens. Functional variation can arise through genome sequence changes such as (**A**) single nucleotide mutations, insertions, and deletions that occur during replication (in red). Hypermutators have an increased rate of mutation due to defects in mismatch repair, (**B**) loss of heterozygosity (LOH) in diploid species, (**C**) altered gene dosage of relevant alleles can occur through aneuploidy, and (**D**) copy number variation (CNV). (**E)** Fungi are also capable of parasexual recombination through the formation of a heterokaryon [1], the merging of nuclei to form a heterozygous diploid [2], followed by recombination and a return to haploidy [3]. (**F**) Mobile genetic elements and the presence of a mycophage can also alter phenotype. Figure prepared using BioRender.

It is essential to understand the mechanisms and frequencies at which population heterogeneity can arise and, therefore, adaptation after selection [18]. In this review, we will primarily focus on genome-based changes that contribute to fungal diversity [14], which include single nucleotide polymorphisms (SNPs) due to mutation, insertions or deletions (indels) (**Fig 1A**), loss of heterozygosity (LOH) (**Fig 1B**), which can occur through mitotic recombination in diploids organisms, whole or partial chromosome duplications through aneuploidy (**Fig 1C**), copy number variations (CNVs) (**Fig 1D**), and parasexual cycles that yield heterokaryons (**Fig 1E**) upon the merging of nuclei to form a heterozygous diploid followed by recombination before a return to haploidy. The movement of mobile elements or loss of a mycophage is another way that populations can become heterogeneous (**Fig 1F**) [19,20]. Other ways in which fungal populations increase diversity include morphological differentiation, stable phenotypic switches, and changes to chromatin state, and these nongenetic changes have been shown to be influenced by mutations that arise and appear to be under selection in vivo as discussed below.

### Evolution through mutation

SNPs and indels in the genome arise from errors in replication. Increases in mutated alleles within a population can occur through direct selection or “hitchhiking” with beneficial mutations. High frequencies of mutated alleles can also arise by genetic drift, which might occur upon the migration of cells to a new site of infection or a substantial reduction in the population size, perhaps due to antibiotic treatment. Mutation rates vary between fungal species and may not be consistent across the genome [21]. Mutations have been predicted to occur at a rate of 1.2 × 10^−10^ per base pair per generation in *Candida albicans* and 0.33 × 10^−9^ per base pair per generation in *Saccharomyces cerevisiae* [14,22]. It has also been posited that diploidy may accelerate the acquisition of adaptive mutations due to the ability to tolerate mutations in 1 allele of 2 [23,24]. Over weeks, months, or years, the divergence of fungal populations can occur through the accumulation of mutations over successive generations, spatial isolation (e.g., different lung lobes), and both constant and variable selective pressures imposed by the host immune response or therapeutic interventions. Several microevolution studies demonstrate polymorphisms that increase fitness and phenotypic diversity in *Candida*, *Aspergillus*, and *Cryptococcus* [21,25,26]. For example, examination of the genomes from 20 clinical isolates of *Clavispora* (*Candida*) *lusitaniae* from respiratory samples from a single individual found between 24 and 131 SNPs between any 2 isolates and significant phenotypic heterogeneity [27]. A study of oral *C*. *albicans* isolates from 8 healthy individuals identified an average of approximately 300 to approximately 1,400 SNP differences between isolates from the same sample, with most of the SNP differences due to LOH events at the gene, region, or chromosome levels [28].

Elevated mutation rates can enable rapid adaptation to local environments. In prominent human fungal pathogens, such as *C*. *neoformans*, *A*. *fumigatus*, *C*. *albicans*, and *Candida glabrata,* hypermutators have been shown to emerge during infection and contribute to treatment failure [29–32]. The hypermutator phenotype often arises from defects in DNA proofreading and repair. For example, defects in Msh2, a core component of the DNA mismatch repair pathway, can increase the rate of acquired resistance to 5-fluoroorotic acid (5-FOA), which selects for uracil auxotrophs, by up to 6.6-fold [33,34]. One study using whole genome sequencing found hypermutators with defective Msh2 in recurrent *C*. *neoformans* infections of people with HIV/AIDS in sub-Saharan Africa [35]. In one lineage, increased diversity resulted from mutations in genes *MSH2*, *MSH5*, and *RAD5*, which encode mismatch repair proteins and conferred a hypermutator phenotype through defects in the repair of base-base and single insertion/deletion mismatches, respectively. Isolates in the Msh2 hypermutator lineage accumulated over 300 mutations daily, while members of nonmutator lineages of recurrent isolates acquired approximately 12 mutations per day [35]. In clinical bloodstream isolates of *C*. *glabrata*, mutations in *MSH2* were found in more than 50% of isolates, indicating that a hypermutation phenotype is advantageous in vivo [30]. Mutations in DNA polymerase Pol3 can also increase the mutation rate in *C*. *neoformans* [36]. The observed isolates in recurrent *C*. *neoformans* infection and *C*. *glabrata* highlight the impact of hypermutation on the genetic basis of infection, providing valuable insights into rapid mutation rates that result in the rapid acquisition of secondary mutations that can improve fitness. The presence of hypermutators accelerates the development of genetic diversity. It can significantly contribute to the emergence of drug-resistant strains, demonstrating the dynamic landscape of chronic fungal infections shaped by the accumulation of mutations.

### Phenotypic changes resulting from the loss of heterozygosity

In diploid fungi, genetic variation in populations can be generated through the LOH due to diverse processes including double strand break-induced repair mechanisms, chromosome nondisjunction, and gene conversion (see A. Gusa and S. Jinks-Robertson [37] for review). Resultant phenotypic changes in newly homozygous strains may be more fit in specific environments. In response to infection-relevant stress conditions such as increased temperature, oxidative stresses, and antifungals, *C*. *albicans* increases the rate at which it undergoes mitotic recombination, resulting in more LOH [38]. Consistent with the idea that physiological signals promote recombination rates, phenotypic and genotypic diversity was found to arise as much as 3 orders of magnitude more rapidly in vivo compared to rates observed in vitro [39,40]. Furthermore, in these studies, rates of recombination were similar in oral and disseminated candidiasis models. In a study of 9 *C*. *albicans* clinical isolates collected from a bone marrow transplant patient over 35 days, fluconazole resistance rapidly developed in multiple isolates after 17 days of treatment with fluconazole and amphotericin B [41]. One mechanism for the increased azole resistance was increased *CDR1* expression through a LOH event that resulted in 2 alleles encoding a hyperactive variant of the *CDR1*-regulator Tac1. Notably, these mutations were not fixed within the population, and different genotypes predominated in various sites in the host. Other studies have also reported azole-resistant clinical isolates acquired through homozygosity of *TAC1* hyperactive alleles, which conferred an intermediate, codominant phenotype when expressed alongside the nonhyperactive allele in a paired azole-sensitive strain [42].

Similar gain-of-function mutations followed by LOH were reported in a study by Dunkel and colleagues [43], where mutations in the gene encoding the drug efflux regulator Mrr1 were identified by comparing fluconazole-resistant isolates of *C*. *albicans* to related susceptible isolates from the same patients. Five of 7 resistant isolates had become homozygous for the mutated allele, which increased resistance to azole antifungals to a level greater than observed in strains that only had a single copy. The acquisition of the Mrr1 heterozygous mutation facilitated the modulation of the variant allele’s gene dosage through LOH in conditions where it was selectively advantageous. The retention of alleles with different levels of activity may circumvent significant tradeoffs for Mrr1 hyperactivity [44,45], which we will discuss further below, such as fitness costs in the absence of a drug [46].

### Aneuploidy and copy number variation rapidly diversify a population through gene dosage

Aneuploidy and CNV are 2 fascinating phenomena that contribute to diversity within fungal populations by modifying the dosage of genes through the gain or loss of whole chromosomes or specific genome regions. Gene redundancy can also facilitate evolution by allowing cells to harbor native and functional alleles. Alterations in ploidy and copy number can increase in response to stress, and this plasticity can significantly affect fitness and treatment outcomes [47]. In particular, the duplication of genes contributing to azole drug resistance is common and challenging for the clinical management of fungal infections [48–50]. Aneuploidies in *C*. *albicans* confer fitness benefits despite associated costs in various physiologically relevant conditions [51], and studies of recurrent *Candida* infections have identified aneuploidy events that alter antifungal resistance [41,52–56]. Similarly, heteroresistant populations of *C*. *neoformans* are known to have acquired disomic chromosomes in response to fluconazole [57]. Evolution studies of *Candida parapsilosis* have identified karyotypes that promote cross-tolerance to multiple drugs [58] and evolved azole resistance through novel mechanisms such as segmental chromosome duplication in *Candida auris* [59]. Whole chromosome duplications, such as trisomy of chromosomes 5 and 6 in *C*. *albicans*, significantly alter host interaction by conferring commensal-like phenotypes with less stimulation of inflammation and weight loss in the mouse oropharyngeal candidiasis model and reduced adhesion and invasion [60].

In the study by Rhodes and colleagues [35], aneuploidies were detected in 7 of 17 pairs of recurrent *C*. *neoformans* isolates recovered from meningitis patients in addition to hypermutator lineages. Chromosome 12 was frequently aneuploid, potentially increasing the expression of *SFB2*, a proposed member of the sterol regulatory element binding protein pathway, and alcohol dehydrogenase-encoding *GNO1*, both genes potentially promoting *C*. *neoformans* virulence. *ERG11*, which encodes a component of the sterol synthesis pathway and the target for azole drugs, also had increased copy numbers in 7 pairs of isolates [35] and was observed even in the absence of drug treatment. These observations underscore the potential for aneuploidies to impact multiple phenotypes.

### Heterokaryon formation and other changes in ploidy

Haploid organisms such as *Aspergillus nidulans* and *A*. *fumigatus* can also undergo recombination through diploid heterokaryon formation and the parasexual cycle. The parasexual cycle and accompanying variation in ploidy in *A*. *nidulans* produce haploid recombinants with improved fitness measured by mycelial growth rate [61]. A screen of *A*. *fumigatus* isolates from CF, chronic pulmonary lung disease, and chronic aspergillosis found evidence for diploid formation in vivo but notably did not find any diploids in 368 acute infection or environmental isolates, suggesting that chronic infections provide a niche environment for genetic diversity to develop through parasexual recombination [62]. Further study is required to understand what stresses in chronic infection stimulate parasex and whether the parasexual process is relevant for other fungi in chronic illness.

Massively polyploid *C*. *neoformans*, which become enlarged in the initial stages of pulmonary infection, are known as “titan cells” and provide an extreme example of changes in ploidy associated with a morphological transition, leading to the variegation of function during infection [63]. Titan cells can be 5 to 10 times larger than normal haploid *C*. *neoformans* cells and make up to 20% of the cell population and increase relative to normal yeast cells throughout infection [64,65]. Exposure to reactive nitrogen species, bacterial cell wall components, etc., can trigger the formation of polyploid titan cell populations in *C*. *neoformans*, which are highly heterogeneous in size and nuclear content [66]. Titanization increases resistance to reactive oxygen species and azoles and induces a strong Th2 response while being resistant to phagocytosis [63]. In clinical isolates obtained from HIV/AIDS patients, these diverse *C*. *neoformans* cells are also accompanied by “seed” cells of diminished cell wall thickness and volume [67]. *Cryptococcus* seed cells are induced by phosphate supplementation and excel at dissemination and invasion of extrapulmonary organs, which is partially dependent on size [67]. The specific role of seed cells compared to other cryptococcal microcell variants in host interaction is unclear. Still, their negative correlation to acute symptoms indicates that they are a relevant morphology in persistence and chronic infection and further highlights the importance of phenotypic heterogeneity in infection.

### Nongenetic mechanisms for the generation of population heterogeneity: *C*. *albicans* as an example

**Fig 2:**
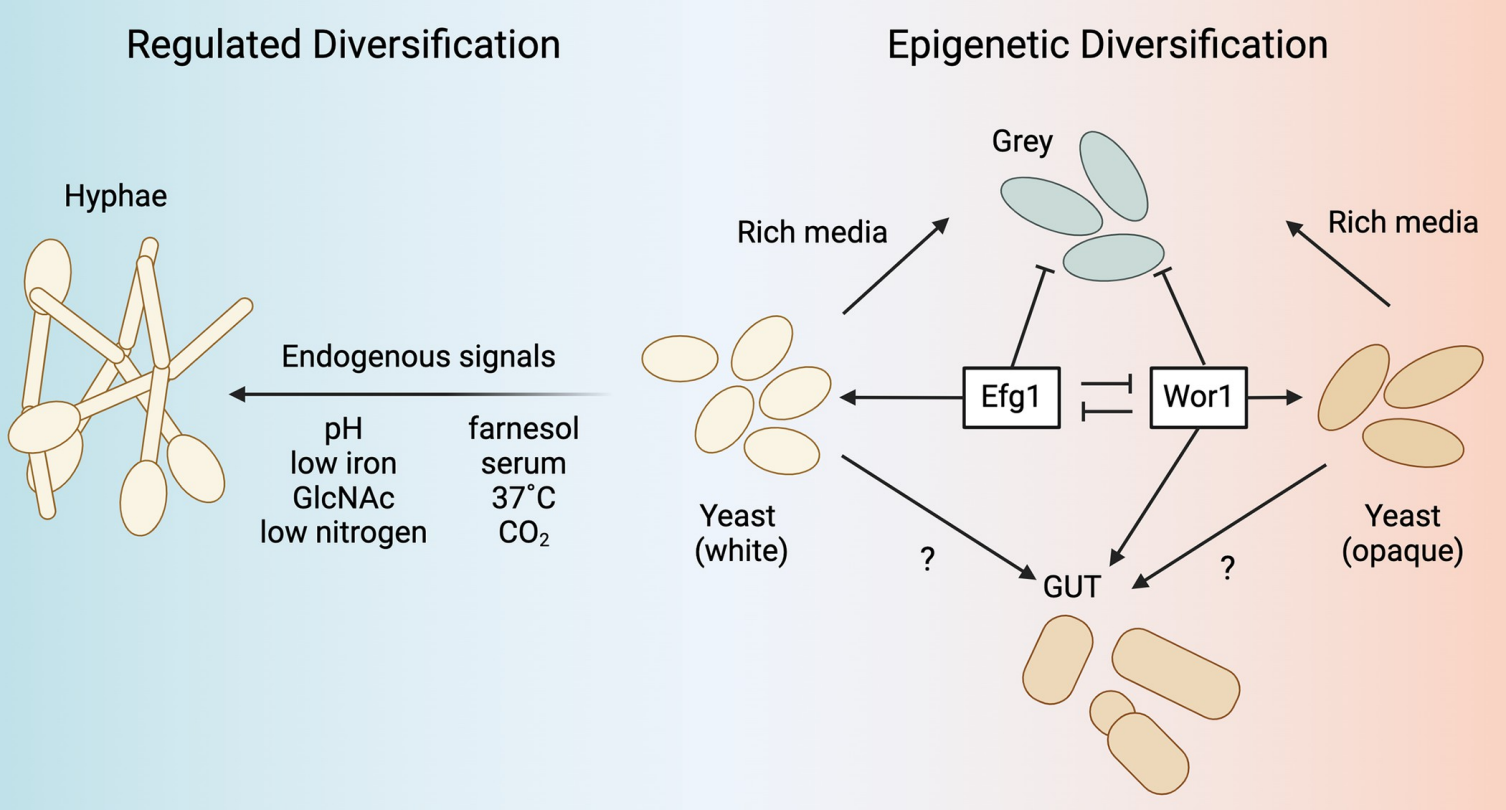
Nongenetic mechanisms that diversify fungal populations: *C*. *albicans* as an example. In *C*. *albicans*, morphological transitions (e.g., the yeast-to-hypha and hyphae-to-yeast transitions) and epigenetic phenotypic switches are driven by transcription factors Efg1 and Wor1. These transitions diversify cell types, such as white, opaque, and grey cell types, that have distinct properties. GlcNAc, N-acetyl-glucosamine; GUT, Gastrointestinally indUced Transition cells. Figure prepared using BioRender.

As detailed above, phenotypic switching, observed in many fungi such as *C*. *neoformans*, enables the coexistence of variable cell states, maximizing a fungus’ ability to occupy multiple metabolic and immunological niches. *C*. *albicans* utilizes particularly well-studied mechanisms of genetic regulation and epigenetic stochasticity to switch between myriad cell states, thoroughly reviewed by Noble and colleagues [68]. First, yeast-to-hyphae transitions contribute to niche specification in *C*. *albicans*. Yeast and hyphal forms of *C*. *albicans* are frequently observed to coexist in biofilms formed in infection [69], and the inability to interconvert between morphologies renders *C*. *albicans* avirulent [70,71]. While filamentation is regulated by a network of transcription factors that respond to factors in the infection environment, genetic changes such as aneuploidy [72] and mutation [73,74] can modulate this response. Second, *C*. *albicans* can stably grow in a variety of distinct cell states named after the appearance of colonies formed by each type (**Fig 2**). The white-opaque switch is a reversible and stochastic transition between 2 cellular states with distinct genetic programming. White and opaque cell types are phenotypically distinct, with a metabolic bias towards glycolysis in white cells and increased beta-oxidation and respiration in opaque cells [75]. The impact of these metabolic differences on *C*. *albicans* fitness is extensively dissected in a 2016 paper by Ene and colleagues [76], which establishes that while white cells have competitive fitness advantages in most tested growth conditions, there are specific substrates (e.g., glucose and triglycine) where opaque cells have improved growth and biofilm formation relative to white cells. More recent work has revealed additional plasticity in the white-grey-opaque and white-GUT switches [77,78]. Homozygous deletion of the white-opaque master regulator Wor1 and transcriptional regulator Efg1 results in the production of semi-mating competent grey cells, while Wor1 overexpression produces the GUT morphology. Various morphotypes are proposed to be an essential component of host–*Candida* interactions in the gut. The central transcriptional regulator of *C*. *albicans* morphology, Efg1, is observed to vary between high and low expression in gut isolates [79,80]. Several recent studies have reinforced the fitness trade-offs of invasive growth in exchange for commensalism, such as increased colonization fitness in *C*. *albicans* strains lacking *SAP6* or *UME6* [81], and loss-of-function mutations in *FLO8* and *EFG1*, which encode hyphal growth regulating transcription factors. The loss-of-function mutations followed by LOH resulted in commensal phenotypes [82,83]. Anderson and colleagues [84] observed extensive diversity within a single host and phenotypic variation between closely related isolates in a study of *C*. *albicans* commensal gut isolates from 35 healthy donors. These studies highlight that during a shift to pathogenesis due to a change in host protective mechanisms, there is diversity in the population, which will likely undergo selection for those strains with maximal fitness. The dynamic nature of fungal morphology and cell state suggests infectious populations exist in heterogeneous cell states, which appears to maximize the occupation of multiple metabolic and immunological niches.

### Assessment of the environmental drivers of selection in chronic, heterogeneous cystic fibrosis fungal populations

The study of CF lower airway infections has provided unique insight into fungal heterogeneity within chronic infections. CF respiratory infections occur in a dynamic neutrophilic environment where the host’s immune defenses continuously interact with colonizing fungal and bacterial pathogens. The study of intraspecies heterogeneity in CF has primarily focused on the most common infections caused by bacteria, such as *Pseudomonas aeruginosa* and *Staphylococcus aureus* [85–87]. In *P*. *aeruginosa*, mutations that lead to amino acid auxotrophies, overproduction of the exopolysaccharide alginate, loss-of*-*function of the LasR quorum sensing regulator, and drug resistance are common. In *S*. *aureus*, mutations in *agr*, which encodes a quorum sensing regulator, or heme auxotrophy, and the small colony variant morphology are common.

**Fig 3:**
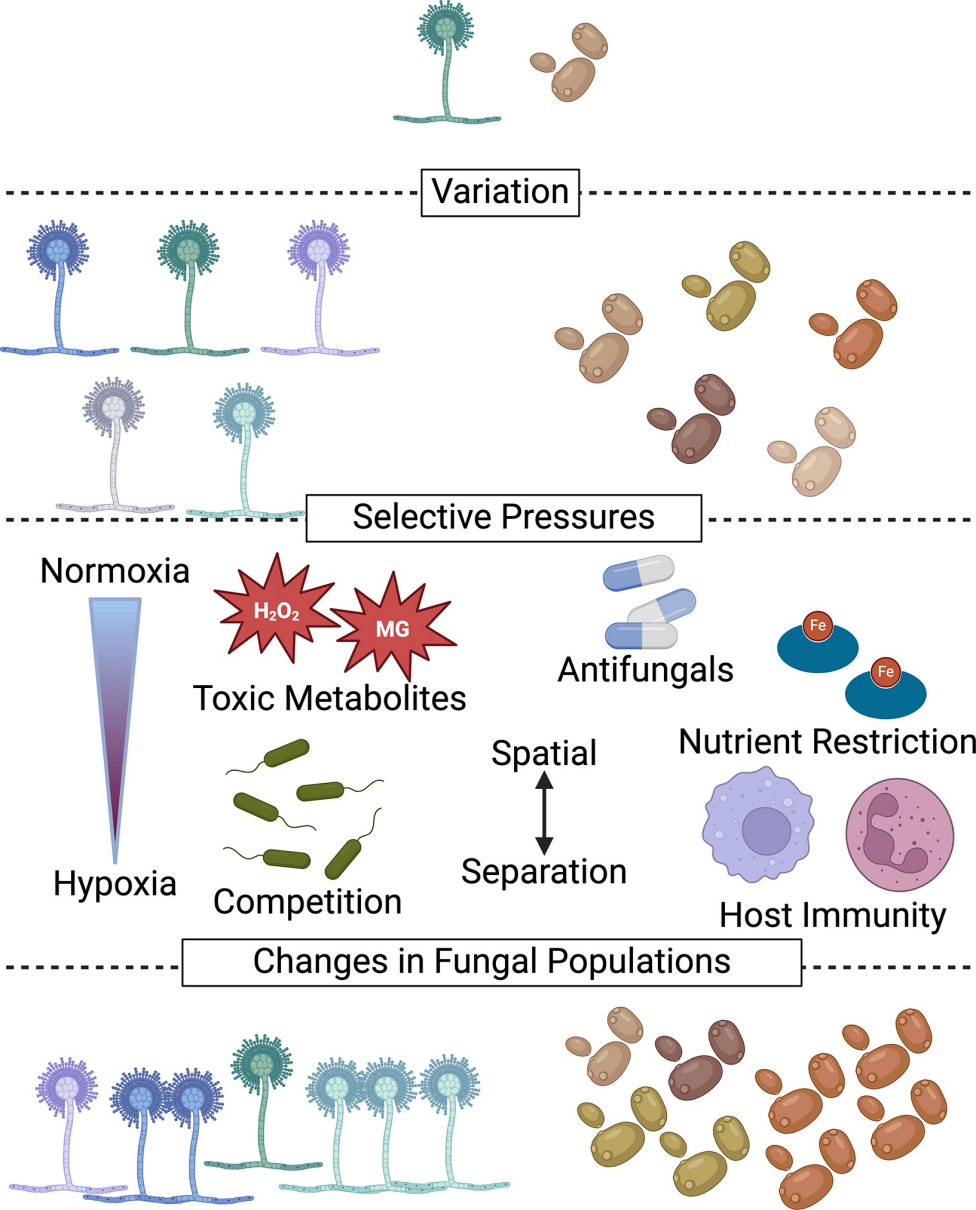
Factors contributing to genetic and phenotypic heterogeneity in fungal populations in CF-associated lung infections. Initial colonizers generate genotypic and phenotypic diversity (variation) through the mechanisms described in Fig 1. Selective pressures include (1) hypoxia, (2) damaging toxic metabolites including hydrogen peroxide (H_2_O_2_) and methylglyoxal (MG), (3) products from other coinfecting microbes, (4) interactions with the immune effectors, (5) nutritional immunity factors that, for example, restrict access to transition metals such as iron (Fe) or zinc, and (6) antifungals or other therapeutic agents. Spatial separation across lungs and lobes of the lung and the dynamic infection environment with periods of disease stability and exacerbation increases the heterogeneity of the population. Figure prepared using BioRender.

In contrast, less is known about how fungi evolve in CF airways. The prevalence of fungi in CF respiratory samples has risen in recent years, and greater diversity in fungal species has been detected [88]. Multiple species of *Candida*, as well as *A*. *fumigatus* and *Exophiala dermatitidis,* are among the most common fungi associated with chronic CF lung infections [89]. While the pathogenicity of these organisms in CF is still an active area of research, there is evidence that fungal colonization contributes to worsened patient outcomes, as highlighted in several papers and reviews [88,90–94]. Below, we will discuss insights into the evolution of fungal populations over time derived from studying the heterogeneity of fungi in CF. These findings contribute to a better understanding of factors influencing infecting microbes, such as host immune responses, antimicrobial treatments, metabolite production, nutrient limitation, and oxygen availability (**Fig 3**).

### *C*. *albicans* mutations in polymicrobial CF lung infections

In work that assessed mycobiome heterogeneity in 28 study participants with CF (pwCF) Kim and colleagues [74], significant heterogeneity in antifungal drug resistance was observed in both *Candida* spp. and *A*. *fumigatus* isolates collected from CF sputum. In 5 pwCF colonized with *C*. *albicans* and 1 colonized by *C*. *parapsilosis*, isolates that had a wrinkled morphology, which is associated with filamentation, were found. Genetic analysis found evidence that the *Candida* populations in each of these 6 individuals had at least 1 lineage with an independent loss-of-function mutation in the gene encoding Nrg1, a well-characterized repressor of filamentation. The repeated loss of Nrg1 function across different individuals and in 2 species suggests that Nrg1 loss-of-function confers increased fitness in the CF lung. Recently, Gnaien and colleagues [73] also used whole genome sequence data to show that *C*. *albicans* from individuals with CF were clonal and that there was clear divergence among isolates from the same patient. Their work found a gain-of-function mutation in the gene encoding Rob1, a positive regulator of filamentation [73]. Because the mutations in *NRG1* and *ROB1* found in CF *C*. *albicans* isolates lead to increased growth as hyphae rather than as yeast, it is reasonable to hypothesize that either the hyphal morphology or genes coregulated with this morphological change (e.g., increased expression of adhesins and host-damaging toxins such as candidalysin or increased acquisition of micronutrients such as iron [95,96]) may promote fitness in chronic lung infections. *C*. *albicans* is frequently found in coinfections with the bacterium *P*. *aeruginosa* [97], and all but one of the hyperfilamentous isolates were recovered from patients coinfected with *P*. *aeruginosa*. One of the C. albicans *nrg1* mutants was isolated from a coinfection with *Burkholderia multivorans* [74]. Interestingly, both the *nrg1* and *rob1* alleles found in CF isolates better resisted the repression of filamentation and antagonism by *P*. *aeruginosa* in in vitro cocultures [73,74,98].

### Dynamic selection for and against high Mrr1 activity in CF *C*. *lusitaniae* infections

Analysis of regional microbial populations by bronchoalveolar lavage found 3 individuals with CF with infections dominated by *C*. *lusitaniae* with no evidence of coinfecting bacteria [99,100]. *C*. *lusitaniae* is a non-*albicans Candida* species closely related to *C*. *auris* and has been observed to rapidly evolve antifungal resistance through *ERG3* and *FKS1* mutations in response to clinical antifungal treatment [101,102]. *C*. *lusitaniae* has been previously identified in CF lung infections [103–105], but its presence in the CF lung is uncommon. In the *C*. *lusitaniae* population from 1 patient, multiple independent mutations in the *MRR1* gene that encodes the multidrug resistance transcription factor were identified, with 13 alleles found within the genomes of the 20 sequenced isolates. Genetic heterogeneity in *MRR1* led to phenotypic variation in azole resistance [27], and the presence of fluconazole-resistant strains was surprising as there was no evidence of prior exposure to antifungals. Thus, it was proposed that the increased Mrr1 activity was under selection for different reasons, such as improved resistance to antimicrobial peptides such as histatin 5 or *P*. *aeruginosa*-produced phenazines through elevated levels of the Mdr1 efflux pump [27,106]. Studies of the Mrr1 regulon also found that it regulates 2 genes encoding methylglyoxal (MG) reductases, Mgd1 and Mgd2, which detoxify MG, an electrophilic, toxic metabolite [107]. Given the impaired detoxification of MG in the CF lung [108,109], it is possible that elevated MG levels contributed to the selection of strains with high Mrr1 activity. The retrospective detection of fluconazole-resistant strains with hyperactive Mrr1 alleles explained the observed azole treatment failure [44]. Interestingly, isolates with *MRR1* alleles with activating mutations frequently acquired secondary nonsense or missense mutations in the 3′ end of the *MRR1* gene, which reduced the constitutive activity of Mrr1 and thus lowered fluconazole and MG resistance [44]. Phenotype analysis of strains with different *MRR1* alleles found that while high Mrr1 activity conferred increased protection against MG, it rendered cells more sensitive to oxidative stress [44]. Furthermore, longitudinal analyses of respiratory samples revealed an inverse correlation between high Mrr1 activity (fluconazole and MG resistance) and hydrogen peroxide resistance at the population level over time, suggesting that there could be opposing selective pressures in the lung [44]. This complex scenario highlights the potential need for combinatorial treatments in addressing drug resistance in CF-associated fungal infections, informed by trade-offs in fitness that commonly accompany specific mutations and phenotypes.

### Mutations in *MRS4* across fungal species in CF lung infections

In all 3 chronic CF *C*. *lusitaniae* infections, at least 1 lineage with loss-of-function mutations in the gene encoding the mitochondrial iron importer Mrs4 was detected [100]. Allelic exchange of the mutated *mrs4* alleles into a common background demonstrated that they all produced a hyperactive iron-scavenging response [100]. Expression of genes involved in iron uptake through heme, siderophores, and reductive mechanisms was significantly increased, and isolates with mutated *mrs4* alleles had significantly greater intracellular iron content than those with fully functional Mrs4. Pooled sequencing of bronchoalveolar lavage fluid from each patient with *C*. *lusitaniae* revealed that different genotypes of *MRS4* were spatially biased to different lobes of the lung, indicating the role of spatial separation in the generation of genetic diversity and the value of studying populations from various locations [100]. Importantly, analysis of isolates of the black yeast *E*. *dermatitidis* from an independent chronic CF infection also showed diversification over time with the persistence of 2 major subpopulations, with evidence for the evolution of a loss-of-function *mrs4* allele in 1 clade [110]. It is speculated that the driving force for the selection of *mrs4* loss-of-function mutations is to overcome nutritional immunity, wherein vital micronutrients such as iron are sequestered by calprotectin and other iron-binding proteins like ferritin and lactoferrin [111–115]. There is evidence for metal restriction in the CF environment, likely due to nutritional immunity factors, even though some studies have presented evidence for increased iron content in sputum and BAL fluid [116,117]. While the loss of Mrs4 function in CF isolates may aid in iron acquisition, an *mrs4Δ/Δ* mutant results in loss of virulence in a *C*. *albicans* murine model of systemic candidiasis [118]. This contradiction highlights that acute and chronic infections require different fitness traits. Further, these studies suggest that changes in iron acquisition and perhaps storage increase fitness in chronic CF-related lung infections in diverse species (e.g., *C*. *lusitaniae* and *E*. *dermatitidis*).

### *A*. *fumigatus* heterogeneity in the CF lung: Changes in Hog1

*A*. *fumigatus* is a ubiquitous mold and a critical causal agent of invasive and chronic infections. The detection of *Aspergillus* sp. in CF clinical samples is associated with worse clinical outcomes and increased lung damage [119]. Ross and colleagues [120] highlight the striking genotypic and phenotypic diversity of *A*. *fumigatus isolates* collected throughout 4 and a half years from a single individual with CF who had not received antifungal treatment. Whole genome sequencing analysis of these longitudinal *A*. *fumigatus* CF isolates identified 2 persistent lineages with differences in phenotypes relevant to CF lung infections, including their ability to grow in low oxygen and to adapt to osmotic and oxidative stress and their sensitivity to voriconazole. One lineage of persistent isolates in this individual contained a novel allele encoding the high-osmolarity glycerol (HOG) pathway protein kinase kinase Pbs2 [120]. Allelic exchange experiments demonstrated that the novel *pbs2* allele encoded a variant that hyperactivates that HOG Map kinase ortholog SakA and is sufficient to confer changed phenotypes in low oxygen, the presence of voriconazole, and high osmolarity conditions. While the novel *pbs2* allele was beneficial in these CF-relevant conditions, it also led to significant differences in morphotype (conidia versus hyphae) specific growth and disadvantages in growth in normoxic environments compared to isolates containing the more common *pbs2* allele. Interestingly, the introduction of the novel *pbs2* allele into more genetically distant backgrounds of *A*. *fumigatus* was not sufficient to promote a growth advantage in CF-relevant environments, highlighting that the benefit of some adaptive mutations can be highly dependent on specific genetic backgrounds and may involve complex genetic interactions.

### Outstanding questions and concluding remarks

Due to their complex and heterogeneous nature, chronic fungal infections are challenging to manage and treat. Genetic heterogeneity in chronic fungal infections is generally not evaluated diagnostically, and the lack of such information may limit the use of therapeutic strategies suited to combat complex populations and communities, such as combination drug therapy [16]. With the increased availability of technology for next-generation sequencing of multiple isolates or populations from clinical samples, it is now possible to describe fungal populations within samples and how they change over time. Current knowledge of the diversity of fungal infection populations in other types of mycoses that occur in diverse body sites, such as the sinuses, mouth, lungs, groin, and feet, is limited. There is evidence that vulvovaginal candidiasis isolates and isolates from aspergillomas can be closely related but genetically distinct, which may be due to genetic adaptation to the host environment [121–123].

The mechanisms driving fungal heterogeneity are varied and often include mutations, mobile elements, LOH, and changes in the copy number of genomic regions or, in some cases, whole chromosomes. Emerging research on mycophage’s prevalence, retention, and effects on human fungal pathogens may add to our understanding of fungal population dynamics. Cross-sectional and longitudinal characterization of fungal populations from CF lung infections found evidence of selection for increased growth in the filamentous morphology, efflux pump activity, iron acquisition, and hypoxia tolerance, among other phenotypes. These examples highlight how studying intraspecies heterogeneity can elucidate relevant host stresses and pathoadaptive mechanisms for clinically important fungi. However, several crucial questions remain unanswered, including the following:

- Are there in vivo or in vitro models that best represent the selective pressures present in chronic infections?
- Are there selective pressures that are specific to chronic infections and acute infections?
- Are there mutations or levels of population heterogeneity predictive of virulence, potential for clearance by immune responses, or the likelihood of antifungal therapy failure?
- Do genetically diverse populations have emergent properties due to interactions between evolved lineages that may influence host damage or response to antifungal action?
- Are body sites distinct in adaptation, or are there convergent phenotypes that repeatedly arise?
- Do other microbes influence the evolution of fungi in complex multispecies infections?
- Are similar pathways undergoing selection across different types of microbial pathogens?

As studies on fungal populations in chronic infections advance, ecological and evolutionary models incorporating parameters such as mutation rate, growth rate, population size, and interactions may be helpful in assessing the strength of different selective pressures. In the future, we hope these insights will aid in developing therapeutic strategies that address the diverse and evolving nature of chronic fungal infections and may ultimately improve outcomes for individuals grappling with these persistent health challenges.
